# Diplotypes of CYP2C9 gene is associated with coronary artery disease in the Xinjiang Han population for women in China

**DOI:** 10.1186/1476-511X-13-143

**Published:** 2014-09-02

**Authors:** Zhenyan Fu, Qing Zhu, Yitong Ma, Ding Huang, Shuo Pan, Xiang Xie, Fen Liu, Erdenbat Cha

**Affiliations:** Department of Cardiovascular Medicine, First Affiliated Hospital of Xinjiang Medical University, Li Yu Shan South Road 137, Urumqi, 830054 China

**Keywords:** CYP2C9, Single-nucleotide polymorphism, Haplotype, Diplotype, Case–control study

## Abstract

**Background:**

Cytochrome P450 (CYP) 2C9 is expressed in the vascular endothelium and metabolizes arachidonic acid to biologically active epoxyeicosatrienoic acids (EETs), which have the crucial role in the modulation of cardiovascular homeostasis. We sought to assess the association between the human CYP2C9 gene and coronary artery disease (CAD) in Xinjiang Han Population of China.

**Methods:**

301 CAD patients and 220 control subjects were genotyped for 4 single-nucleotide polymorphisms (SNPs) of the human CYP2C9 gene (rs4086116, rs2475376, rs1057910, and rs1934967) by a Real-Time PCR instrument. The datas were assessed for 3 groups: total, men, and women via diplotype-based case–control study.

**Results:**

For women, the distribution of genotypes, dominant model and alleles of SNP2 (rs2475376) showed significant difference between the CAD patients and control participants (p = 0.033, P = 0.010 and p = 0.038, respectively). The significant difference of the dominant model (CC vs CT + TT) was retained after adjustment for covariates in women (OR: 2.427, 95% confidence interval [CI]: 1.305-4.510, p = 0.005). The haplotype (C-T-A-C) and the diplotypes (CTAC/CTAC) in CYP2C9 gene were lower in CAD patients than in control subjects (p* = 0.0016, and p* = 0.036 respectively). The haplotype (C-C-A-T) was higher in the CAD patients than in the control subjects in women (p* = 0.016).

**Conclusions:**

CC genotype of rs2475376 and C-C-A-T haplotype in CYP2C9 may be a risk genetic marker of CAD in women. T allele of rs2475376, the haplotype (C-T-A-C) and the diplotype (CTAC/CTAC) could be protective genetic markers of CAD for women in Han population of China.

## Introduction

CAD is a complex multifactorial and polygenic disorder thought to result from an interaction between an individual's genetic makeup and different environments [1]. Increasing evidence from animals and clinical and epidemiological studies has repeatedly supported the likelihood of a genetic contribution to CAD susceptibility [2, 3]. Cytochrome P450 (CYP) genes is a super family of cysteine-heme enzymes, which catalyze the oxidation of various drugs and endogenous substrates, such as vitamin D, steroids, and fatty acids, including arachidonic acid (AA) [4]. CYP enzymes of the P450 2C9 subfamily are found in the liver, vascular smooth muscle, endothelial cells of human aorta and coronary artery [5–7]. In human liver, CYP2C9 is responsible for 50% of the epoxygenase activity, and metabolizes a wide variety of clinically important drugs, including losartan and S-warfarin [8, 9]. In human heart, CYP2C9, as well as CYP2C8 and CYP2J2, participates in metabolizing arachidonic acid to epoxyeicosatrienoic acids (EETs) [10, 11]. EETs are supposed to play the key role in endothelial cell homeostasis, showing protective vascular effects including vasodilatation, anti-inflammtory, anti-apoptotic and anti-thrombotic effects [12–14]. They are also involved in myocardial preconditioning and have cardioprotective effects by increasing postischemic function and reducing myocardial infarct size [15, 16]. Genetic polymorphisms might affect the activity of EETs, which determine susceptibility to the development of CAD. In recent years, many studies have shown the polymorphisms of CYP2C9 gene (rs1057910) were associated with the cardiovascular risk [17, 18]. Given this background, we sought to investigate the possible association between the genetic variation of CYP2C9 and CAD in Xinjiang Han population of China.

## Methods

### Ethical approval of the study protocol

This study was approved by the Ethics Committee of the First Affiliated Hospital of Xinjiang Medical University (Urumqi, China). It was conducted according to the standards of the Declaration of Helsinki. Written informed consent was obtained from all participants. All participants explicitly provided permission for DNA analyses as well as collection of relevant clinical data.

### Subjects

All patients and controls were enrolled from the First Affiliated Hospital of Xinjiang Medical University (Urumqi, China) from January 2011 to April 2013. The study involved 301 patients with CAD defined as the presence of at least one significant coronary artery stenosis of more than 50% luminal diameter on coronary angiography. 220 Control participants did not have coronary artery stenosis and did not show clinical or electrocardio-graphic evidence of myocardial infarction (MI) or CAD. Data and information about traditional coronary risk factors, including hypertension, diabetes mellitus (DM), and smoking, were collected from all study participants. The diagnosis of hypertension was established if patients were on antihypertensive medication or if the mean of 3 measurements of systolic blood pressure (SBP) >140 mmHg or diastolic blood pressure (DBP) >90 mmHg, respectively. Diabetes mellitus was defined by fasting plasma glucose >7.0 mmol/L and also if patients were taking antidiabetic medication or insulin therapy. “Smoking” was classified as smokers (including current or ex-smokers) or non-smokers. All patients with impaired renal function, malignancy, connective tissue disease, or chronic inflammatory disease were excluded.

### Blood collection and DNA extraction

Blood samples were taken from all participants. The blood samples were drawn into a 5 ml ethylene diamine tetraacetic acid (EDTA) tube and centrifuged at 4000 × g for 5 min to separate the plasma content. Genomic DNA was extracted from the peripheral leukocytes using standard phenol–chloroform method. The DNA samples were stored at -80°C until use. When used, the DNA was diluted to 50 ng/ul concentration.

### Genotyping

**Figure 1:**
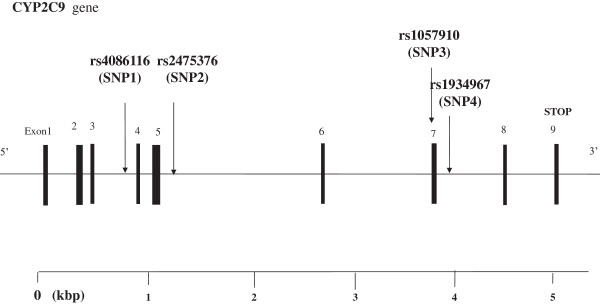
**Structure of the human CYP2C9 gene. This gene consists of 9exons separated by 8 introns.** Boxes indicate exons, and lines indicate introns and intergenic regions. Filled boxes indicate coding regions. Arrows mark the locations of polymorphisms.

There are 1375 SNPs for the human CYP2C9 gene listed in the National Center for Biotechnology Information SNP database (http://www.ncbi.nlm.nih.gov/SNP). Using the Haploview 4.2 software and the HapMap phrase II database, we obtained four tag SNPs (rs4086116, rs2475376, rs1057910 and rs1934967) by using minor allele frequency (MAF) > =0.01 and linkage disequilibrium patterns with r^2^ > =0.5 as a cut off. We designated these SNPs as SNP1, SNP2, SNP3, and SNP4 (rs4086116, rs2475376, rs1057910 and rs1934967) in order of increasing distance from the CYP2C9 gene 5′end (Figure 1). SNP1, SNP2, and SNP4 are located in intron. SNP3(rs1057910) is located in exon7, and had a non-synonymous substitution amino acid change, which is defined by an A-to-C nucleotide substitution that leads to an exchange of leucine by isoleucine at amino acid position 359.

Genotyping was undertaken using the TaqMan® SNP Genotyping Assay (Applied Biosystems) using Taq amplification, TaqMan® SNP Genotyping Assays were carried out. The primers and probes used in the TaqMan® SNP Genotyping Assays (ABI) were chosen based on information at the ABI website (http://myscience.appliedbiosystems.com). Thermal cycling was done using the AppliedBiosystems 7900HT Fast Real-Time PCR System. Plates were read on Sequence Detection Systems (SDS) automation controller software v2.3 (ABI).PCR amplification was performed using 3.0 μl of TaqMan Universal Master Mix, 0.15 μl probes and 1.85 ddH_2_O in a 6-μl final reaction volume containing 1 μl DNA. Thermal cycling conditions were as follows: 95°C for 5 min; 40 cycles of 95°C for 15 s; and 60°C for 1 min. All 96 wells Plates were read on Sequence Detection Systems (SDS) automation controller software v2.3 (ABI).

### Biochemical analysis

Serum concentrations of total cholesterol (TC), triglyceride (TG), glucose, high-density lipoprotein cholesterol (HDL-C), low-density lipoprotein cholesterol (LDL-C), blood ureanitrogen (BUN), creatinine (Cr) and uric acid were measured using standard methods in the Central Laboratory of the First Affiliated Hospital of Xinjiang Medical University as described previously.

### Statistical analysis

All continuous variables (e.g. age, BMI, cholesterol levels) are presented as means ± standard deviation (S.D.). The continuous variables conform to normal distribution, and the study is a large sample data, so the differences of all continuous variables between the CAD and the Control groups were analyzed using T-test**.** The differences in the frequencies of smoking, hypertension, DM, and CYP2C9 genotypes were analyzed using Fisher’s exact test. Chi-square analysis was used to test the deviations of genotype distribution from the Hardy-Weinberg equilibrium and to determine the differences of allele or genotype frequencies between patients and controls. Logistic regression analyses were used to assess the contribution of the major risk factors. And adjusted estimations of conditioned relative risk and 95 % confidence intervals (CIs) were done. All statistical analyses were performed using SPSS 17.0 for Windows (SPSS Institute, Chicago, USA). P-values were considered to be significant at the 0.05 level. Haplotypes were estimated using the expectation maximization algorithm and the software SNPAlyze version 3.2 (Dynacom, Yokohama, Japan), and using the SHEsis platform to verify reliability [19, 20]. The estimated diplotypes (combinations of two haplotypes) in each subject were analyzed using the software SNPAlyze version 3.2 (Dynacom, Yokohama, Japan), The *P* value of haplotype and diplotype were revised by False discovery rate.

## Results

Table 1 showed the clinical characteristics of the CAD patients (n = 301) and control participants (n = 220). For total, men and women subjects, there was no significant difference in age between CAD patients and control subjects. It meant the study was age-matched case–control study. We observed several differences between the two groups of patients. As expected, several common risk factors for CAD were significantly different between the two subgroups: Glu, low HDL-C, high LDL-C, EH, DM. For total, the serum concentrations of glucose (Glu), LDL-C were significantly higher for CAD patients than for control participants (p < 0.05), and the serum concentrations of HDL-C were significantly lower for CAD patients than for control participants (p < 0.05). The prevalence of DM was significantly higher for patients with CAD than for control participants. For men, the serum concentration of LDL-C was significantly higher for CAD patients than for control participants (p < 0.05). The prevalence of EH, DM, and smoking were significantly higher for patients with CAD than for control participants. For women, the prevalence of EH and DM were significantly higher for patients with CAD than for control participants.

**Table 1:** **Characteristics of study participants**

		Total			Men			Women	
	CAD patients	Control subjects	p Value	CAD patients	Control subjects	p Value	CAD patients	Control subjects	p Value
Number (n)	301	220		202	126		99	94	
Age (years)	59.13 ± 8.97	57.64 ± 8.78	0.092	60.73 ± 9.12	58.66 ± 8.43	0.077	62.53 ± 8.45	61.39 ± 7.81	0.364
BMI (kg/m^2^)	25.74 ± 3.39	25.44 ± 3.51	0.353	25.16 ± 5.16	25.46 ± 3.81	0.591	24.68 ± 6.32	25.58 ± 5.01	0.294
Pulse (beats/min)	74.05 ± 10.13	74.13 ± 11.58	0.933	74.34 ± 10.84	74.53 ± 11.04	0.874	73.46 ± 8.52	73.59 ± 12.30	0.937
BUN(mmol/L)	5.46 ± 1.58	5.53 ± 1.63	0.620	5.67 ± 2.12	5.55 ± 1.70	0.627	5.40 ± 1.83	5.36 ± 1.51	0.855
Cr(umol/L)	75.07 ± 20.99	74.09 ± 22.71	0.625	77.50 ± 18.01	76.05 ± 10.00	0.790	74.54 ± 12.90	79.26 ± 10.66	0.762
Glu (mmol/L)	6.10 ± 2.04	5.73 ± 1.91	0.043*	6.03 ± 1.99	5.65 ± 1.92	0.096	6.32 ± 2.53	5.84 ± 1.89	0.139
TG (mmol/L)	1.75 ± 1.02	1.81 ± 1.23	0.545	1.71 ± 0.978	1.79 ± 1.35	0.498	1.94 ± 1.46	1.83 ± 1.01	0.306
TC (mmol/L)	4.09 ± 1.08	4.24 ± 0.98	0.137	4.05 ± 1.70	4.14 ± 1.01	0.595	4.38 ± 1.07	4.38 ± 0.93	0.430
HDL (mmol/L)	1.04 ± 0.37	1.15 ± 0.33	0.001*	1.10 ± 0.94	1.07 ± 0.289	0.737	1.08 ± 0.32	1.26 ± 0.34	0.368
LDL (mmol/L)	2.44 ± 0.77	2..25 ± 0.80	0.004*	2.52 ± 2.20	2.29 ± 1.91	0.007*	2.36 ± 0.80	2.35 ± 0.88	0.461
EH (%)	63.6	51.54	0.072	61.4	48.7	0.032*	65.6	54.1	0.110*
DM (%)	19.95	10.15	0.029*	17.8	8.9	0.043*	22.1	11.4	0.014*
Smoke (%)	21.32	12.56	0.143	37.6	23.0	0.008*	5.05	2.13	0.278

BMI, body mass index; BUN, blood urea nitrogen; Cr, creatinine; Glu, glucose; TG, triglyceride; TC, total cholesterol; HDL, high density lipoprotein; LDL, low density lipoprotein; EH, essential hypertension; DM, diabetes mellitus.

Continuous variable were expressed as mean ± standard deviation. *P* value of continuous variables was calculated by independent T-T test. The P value of categorical variable was calculated by Fisher's exact test. *P>0.05.

Table 2 showed the distribution of genotypes and alleles of SNP1, SNP2, SNP3, and SNP4 of CYP2C9 gene. The genotype distributions for each of the SNPs were in agreement with the predicted Hardy-Weinberg equilibrium values (data not shown). For total, the distribution of the four SNPs genotypes and alleles showed no difference between the CAD patients and control participants. For men, the distribution of the dominant model of SNP2 (rs2475376) (CC vs CT + TT) was higher in CAD patients than in control participants (p = 0.045). For women, the distribution of genotypes, of SNP2 (rs2475376) showed significant difference between the CAD patients and control participants (p = 0.033). The dominant model (CC vs CT + TT) was significantly higher for CAD patients than for control subjects (p = 0.010). The frequency of T allele (rs2475376) was lower for CAD patients than for control subjects (p = 0.038).

**Table 2:** **Genotype and allele distributions in patients with CAD and control subjects**

				Total			Men			Women		
				CAD	Control	p	CAD	Control	p	CAD	Control	p
				n = 301	n = 220		n = 202	n = 126		n = 99	n = 94	
rs4086116	Genotype		C/C	237	176	0.356	158	97	0.464	79	79	0.559
(SNP1)			C/T	56	42		39	28		17	14	
			T/T	8	2		5	1		3	1	
		Dominant model	CC	237	176	0.726	158	97	0.194	79	79	0.444
			CT + TT	64	44		44	29		20	15	
		Recessive model	TT	8	2	0.151	5	1	0.269	3	1	0.338
			CT + CC	293	218		197	125		96	93	
	Allele		C	530	394	0.449	355	222	0.932	175	172	0.312
			T	72	46		49	30		23	16	
rs2475376	Genotype		C/C	104	76	0.834	60	51	0.122	44	25	0.033*
(SNP2)			C/T	142	108		102	56		40	52	
			T/T	55	36		40	19		15	17	
		Dominant model	CC	104	76	0.999	60	51	0.045*	44	25	0.010*
			CT + TT	197	144		142	75		55	69	
		Recessive model	TT	55	36	0.571	40	19	0.279	15	17	0.584
			CT + CC	246	184		162	107		84	77	
	Allele		C	350	260	0.758	222	158	0.051	128	102	0.038*
			T	252	180		182	94		70	86	
rs1057910	Genotype		A/A	237	178	0.282	156	96	0.76	81	82	0.174
(SNP3)			A/C	49	37		37	26		12	11	
			C/C	15	5		9	4		6	1	
		Dominant model	AA	237	178	0.543	156	96	0.829	81	82	0.229
			AC + CC	64	42		46	30		18	12	
		Recessive model	CC	15	5	0.112	9	4	0.563	6	1	0.063
			AC + AA	286	215		193	122		93	93	
	Allele		A	523	393	0.233	349	218	0.965	174	175	0.082
			C	79	47		55	34		24	13	
rs1934967	Genotype		C/C	209	152	0.126	144	79	0.203	65	73	0.161
(SNP4)			C/T	55	61		49	42		6	19	
			T/T	11	7		9	5		2	2	
		Dominant model	CC	209	152	0.933	144	79	0.105	65	73	0.065
			CT + TT	92	68		58	47		34	21	
		Recessive model	TT	11	7	0.77	9	5	0.832	2	2	0.958
			CT + CC	290	213		193	121		97	92	
	Allele		C	499	365	0.978	337	200	0.19	162	165	0.105
			T	103	75		67	52		36	23	

CAD, coronary artery disease.

The *P* value of genotype was calculated by Fisher's exact test.^*^ *P*>0.05.

Table 3 showed that multiple logistic regression analyses were done with or without EH, DM, and smoking. The significant difference of the dominant model (CC vs CT + TT) was retained after adjustment for covariates in women, but not in men (for women, OR: 2.427, 95% confidence interval [CI]: 1.305-4.510, p = 0.005; and for men, OR: 1.372, 95% CI: 0.861-2.186, p = 0.184).

**Table 3:** **Results of Logistic analysis for the dominant model (CC vs CT + TT) of SNP2**

		Total			Men			Women	
	OR	95% CI	p	OR	95% CI	p	OR	95% CI	p
CC vs CT + TT	1.299	0.901-1.873	0.161	1.372	0.861-2.186	0.184	2.427	1.305-4.510	0.005*
EH	1.133	0.798-1.609	0.486	0.981	0.627-1.533	0.932	1.543	0.855-2.785	0.150
DM	1.149	0.671-1.968	0.614	1.095	0.574-2.089	0.783	1.186	0.425-3.313	0.745
Somke	1.307	0.917-1.862	0.139	0.952	0.592-1.530	0.839	2.905	0.536-15.741	0.216

EH, essential hypertension; DM, diabetes mellitus; CAD, coronary artery disease. * p>0.05.

**Figure 2:**
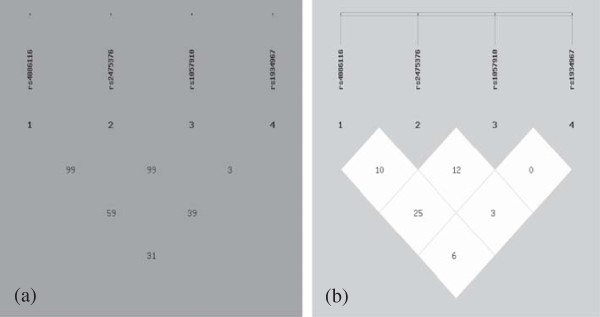
**Pairwise estimates of linkage disequilibrium (LD) between each CYP2C9 polymorphism is plotted for Han population using SHEsis platform.** Each polymorphism is numbered according to its position in the CYP2C9 gene as presented in Figure 1. **(a)** showed **| D'|** and different colors represent different degree of linkage disequilibrium. The darker the color,wasthe stronger the degree of linkage disequilibrium was **(b)** showed r^2.^

Table 4 showed patterns of linkage disequilibrium in the CYP2C9 gene, with their |D′| and r^2^ values. |D′| values from 0.7 to 1 indicate strong LD between a pair of SNPs. |D′| values from 0.25 to 0.7 indicate moderate LD and |D′| values of 0–0.25 indicate low LD. In our study, two strong LD patterns were observed between SNP1 and SNP2 (|D′| = 0.998), SNP2 and SNP3 (|D′| = 0.999). Three moderate LD patterns (|D′| values from 0.25 to 0.7) were observed between SNP1 and SNP3 (|D′| = 0.593), SNP1 and SNP4 (|D′| = 0.311), SNP2 and SNP4 (|D′| = 0.392). In addition, a low LD pattern (|D′| < 0.25) was observed between SNP3 and SNP4 (|D′| = 0.032) (Figure 2). Although LD pattern between SNP3 and SNP4 was low, there were linkage disequilibrium between SNP3 and the two SNPs (SNP1, SNP2), the same as SNP4. We can consider that all four SNPs were located in one haplotype block. R^2^ values of the four SNPs were all <0.5, it means the four SNPs can not replace each other [21, 22]. Then, we use the four SNPs to establish haplotype by the order of SNP1-SNP2-SNP3-SNP4 for all groups.

**Table 4:** **Pairwise linkage disequilibrium (| D'| above diagonal and r** ^***2***^ **below diagonal) for the four SNPs**

		| D'|			
	SNP	SNP1	SNP2	SNP3	SNP4
r^*2*^	SNP1		0.998	0.593	0.311
	SNP2	0.101		0.999	0.393
	SNP3	0.255	0.127		0.032
	SNP4	0.069	0.238	0.001	

**| D'|**

Table 5 showed the distribution of haplotypes in CAD patient and control participants. There were twelve haplotypes established in all subjects. The overall distribution of the haplotypes were significantly different between the CAD patients and the control subjects (all p < 0.0001). The most frequence haplotype in this study was 0100 (C-T-A-C) haplotype. For women, the frequency of C-T-A-C was significantly lower in the CAD patients than in the control subjects (nominal p = 0.0032, adjusted p* = 0.016). In addition, the frequency of the 0001 (C-C-A-T) haplotype was higher in the CAD patients than in the control subjects in women (nominal p = 0.0016, adjusted p* = 0.016). For total and men, the frequency of haplotypes was no diffenerce between the CAD patients and the control subjects.

**Table 5:** **The distubution of haplotype in CAD patient and control participants**

			Total					Men				Women		
			CAD (%)	Control (%)	Nominal p	Adjusted p	CAD (%)	Control (%)	Nominal p	Adjusted p	CAD (%)	Control (%)	Nominal p	Adjusted p
1	0100	CTAC	34.02	37.16	0.2699	0.6747	38.09	34.7	0.2299	0.3831	26.02	41.38	0.0032*	0.016*
2	0000	CCAC	34.47	35.16	0.7979	0.9973	31.87	31.41	0.3916	0.3916	39	40.04	0.3360	0.4200
3	1000	TCAC	2.5	2.48	0.9727	0.9727	1.55	3.04	0.1537	0.3074	4.43	1.73	0.0761	0.1900
4	1100	TTAC	1.22	ˉ	0.0221	0.2210	1.06	ˉ	0.1472	0.3680	1.55	ˉ	0.0902	0.1503
5	0010	CCCC	5.4	4.3	0.4255	0.8510	5.83	6.08	0.3707	0.4119	4.41	1.41	0.0634	0.1133
6	1010	TCCC	2.27	2.34	0.9226	1.0000	2.28	2.89	0.3394	0.4243	2.34	2.02	0.3537	0.3537
7	0110	CTCC	3.9	1.52	0.0300	0.1500	3.75	1.25	0.1020	0.5100	4.06	1.18	0.0877	0.1754
8	0001	CCAT	10.05	9.18	0.5749	0.8212	8.16	13.3	0.1000	1.0000	13.15	3.04	0.0016*	0.016*
9	1001	TCAT	2.49	3.12	0.5333	0.8888	3.88	2.7	0.2834	0.4049	0	3.72	-	-
10	0101	CTAT	0	2.23	-	-	0	1.36	-	-	1.74	3.18	0.1573	0.2242
11	1101	TTAT	2.25	0	-	-	2.08	0	-	-	1.98	0	-	-
12	0011	CCCT	1.43	2.52	0.1746		1.47	3.27	0.1230	0.4100	1.31	1.05	0.3476	0.3862
13	1011	TCCT	-	-	-	-	-	-	-	-	0	1.26	-	-

The p value of haplotype was calculated by Fisher's exact test, and revised by False discovery rate. ^*^ p>0.05;

‘0 represents major allele’ and 1 represents minor allele’.

“0100” refers respectively the major allele of the SNP1,minor allele of the SNP2,major allele of the SNP3,major allele of the SNP4.

The p value of each haplotype by the order of SNP1-SNP2-SNP3-SNP4 is relative to the other haplotypes as a group (overall p <0.0001).

Table 6 showed the distribution of diplotypes in CAD patients and control participants. For women, the two diplotypes (CTAC/CTAC, CTAC/CCAC) in CYP2C9 gene were significantly lower in the CAD patients than in the control subjects (nominal p = 0.004, and p = 0.016 respectively), while after revised by False discovery rate, the diplotypes (CTAC/CCAC) was no difference between CAD patients than the control subjects (adjusted p = 0.072).The homozygous diplotype (CTAC/CTAC) was associated with decreased risk of CAD in women. For total and men, the frequencies of diplotypes were no diffenerce between the CAD patients and the control subjects.

**Table 6:** **The distubution of diplotype of CYP2C9 in CAD patient and control participants**

				Total								Men						Women			
				CHD	Control	OR	95% CI	Nominal p	Adjusted p	CHD	Control	OR	95% CI	Nominal p	Adjusted p	CHD	Control	OR	95% CI	Nominal p	Adjusted p
1	**1/1**	0100/0100	CTAC/CTAC	47	52	1.511	0.893-2.557	0.122	1	40	28	0.824	0.421-1.611	0.334	0.334	7	24	0.251	0.105-0.876	0.004*	0.036*
2	**1/2**	0100/0000	CTAC/CCAC	72	59	0.858	0.576-1.278	0.451	1	53	25	1.437	0.839-2.462	0.149	0.745	19	34	0.419	0.218-0.806	0.016*	0.072
3	**2/2**	0000/0000	CCAC/CCAC	39	24	1.216	0.708-2.088	0.479	0.958	32	10	2.184	1.033-4.615	0.074	0.740	7	14	0.435	0.167-1.130	0.108	0.324
4	**1/3**	0100/0001	CTAC/CCAT	19	18	0.756	0.387-1.477	0.412	1	12	13	0.549	0.242-1.245	0.195	0.488	7	5	1.354	0.414-4.425	0.351	0.526
5	**1/4**	0100/0010	CTAC/CCCC	12	8	1.100	0.442-2.739	0.837	1	9	6	0.933	0.324-2.686	0.326	0.408	3	2	0.438	0.235-8.800	0.308	0.554
6	**1/5**	0100/1001	CTAC/TCAT	10	5	1.478	0.498-4.386	0.479	1	9	3	1.912	0.508-7.201	0.220	0.440	1	2	0.469	0.042-5.264	0.424	0.424
7	**2/5**	0000/1001	CCAC/TCAT	5	4	0.912	0.242-3.437	0.900	0.981	5	0	-	-	-		0	4	-	-	-	
8	**5/5**	1001/1001	TCAT/TCAT	3	1	2.205	0.228-21.338	0.484	0.830	1	1	0.622	0.039-10.032	0.294	0.49	2	0	-	-	-	
9	**1/7**	0100/1000	CTAC/TCAC	6	4	1.098	0.306-3.939	0.891	1	1	2	0.308	0.028-3.437	0.321	0.467	5	2	2.477	0.463-12.931	0.278	0.626
10	**1/8**	0100/1010	CTAC/TCCC	3	3	0.728	0.146-3.642	0.698	1	1	1	0.622	0.039-10.032	0.327	0.363	2	2	0.948	0.131-6.874	0.383	0.431
11	**2/8**	0000/1010	CCAC/TCCC	7	5	1.024	0.321-3.269	0.968	0.968	0	5	-	-	-		7	0	-	-	-	
12	**1/9**	0100/1011	CTAC/TCCT	5	8	0.448	0.144-1.387	0.153	0.918	4	6	0.404	0.112-1.461	0.154	0.513	1	2	0.469	0.042-5.264	0.353	0.454

The p value of diplotype was calculated by Fisher's exact test, and revised by False discovery rate.^*^p>0.05; The odds ratio (OR) and 95 % confidence interval (CI) of each diplotype are relative to the other diplotype as a group. The total diplotypes with very rare count (<3) are not shown. 0 represents major allele and 1 represents minor allele.

## Discussion

Endogenous CYP metabolites such as epoxyeicosatrienoic acids (EETs), hydroxyeicosatetraenoic acids, prostacyclin (PGI2), aldosterone, and sex hormones have been demonstrated to be involved in coronary artery disease, stroke, hypertension, and other cardiovascular diseases [11]. Arachidonic acid can be metabolized by the CYP2C9 subfamily to EETs, which has been established to have five physiological functions. First, EETs produces vasodilation in a number of vascular beds by activating the smooth muscle large conductance Ca^2+^-activated K^+^ channels (BK_Ca_) [6, 14]. Second, EETs may act as endothelium-derived hyperpolarizing factors (EDHF) [16], particularly in the coronary circulation. EDHF possess potent vasodilating effects hyperpolarize vascular smooth muscle cells (VSMCs) by activating K_-Ca_[6, 23, 24]. Third, EETs inhabit inflammation responses by decreasing the cytokine-induced endothelial expression of vascular cell adhesion molecule-1 (VCAM-1) and to decrease leukocytes adhesion to the vascular wall by inhibiting nuclear factor κB (NF-κB) and IκB kinase [25]. Fourth, EETs have antithrombotic effects by inhibiting platelet adhesion to endothelial cells, inhibiting platelet aggregation, and enhancing the expression and activity of tissue plasminogen activator [26]. Fifth, in the kidney, EETs are important regulators of glomerular filtration by activating Na^+^/H^+^ exchanger and mediate pressure natriuresis and long-term control of blood pressure [27, 28]. CYP2C9 polymorphisms might affect the biosynthesis and activity of EETs, which determines susceptibility to the development of CAD. In this study, we hypothesized that variability in CYP2C9 gene might affect the risk of CAD. We genotyped four SNPs of the gene in a Han population, and assessed the association between the CYP2C9 gene and CAD using diplotype-based case–control analyses.

EH and DM were both common risk factors for CAD. As expected, in our study, we found that the prevalence of EH and DM were significantly higher for patients with CAD than for control participants for women. The frequency of T allele of SNP2 (rs2475376) was about 0.409, which was slightly higher than the frequency of T allele of the Chinese Han people (about 0.378) in PubMed database. The distribution of genotypes, dominant model and alleles of SNP2 (rs2475376) showed significant difference between the CAD patients and control participants (p = 0.033, P = 0.010 and p = 0.038, respectively). However, the significant difference of the dominant model (CC vs CT + TT) was retained after adjustment for covariates in women, but not in men (for women, OR: 2.427, 95% confidence interval [CI]: 1.305-4.510, p =0.005; and for men, OR: 1.372, 95% CI: 0.861-2.186, p =0.184). We speculated women carrying CC genotype seem to have a lower ability to synthesize EETs. It means CC genotype of rs2475376 may be an increased risk factor of CAD. The frequency of T alleles (rs2475376) was significantly lower for CAD patients than for control subjects. Women carrying mutant T allele seem to have a higher ability to synthesize EETs. It means that T alleles (rs2475376) may be a decreased risk factor of CAD.

For women, the frequency of the 0100 (C-T-A-C) haplotype was significantly lower in the CAD patients than in the control subjects, and the frequency of the 0001 (C-C-A-T) haplotype was higher in the CAD patients than in the control subjects. Human being is of homologous chromosomes, therefore, diplotype is more convincing than haplotype. For women, the homozygous diplotype (CTAC/CTAC) was significantly lower in the CAD patients than in the control subjects. It means that C-T-A-C haplotype, the homozygous diplotype (CTAC/CTAC) may be decreased risk factors of CAD. C-C-A-T haplotype may be an increased risk factor of CAD. These results of haplotype and diplotype were consistent with the results of CC genotype and T allele of SNP2 (rs2475376).

In our study, we observed a protective effect of the CYP2C9 mutant allele for the development of CAD only in women. For the gender differences, we can explain it by the following reasons. First, this could be attributed to sex hormones. Sex hormones such as estrogens protect against oxidative stress and are known to be vasoprotective [17, 18, 29]. Second, there were some researches [30, 31], which show that estrogens protect the EETs against being hydrolyzed by soluble epoxide hydrolase (sEH). Hence, women carrying mutant T allele seem to have a lower risk for suffering CAD. Third, this may be related to antioxidation of CYP2C9. CYP2C9 has been shown to be a major source of reactive oxygen species (ROS) within coronary artery endothelial cells [32]. Lower formation of oxygen radicals in carriers of mutant alleles might explain our findings.

In addition, many previous studies showed the polymorphisms of CYP2C9 gene (rs1057910) associated with the cardiovascular risk. An increased risk of MI was found in association with CYP2C9 variants (rs1057910) among women [17, 18, 33, 34]. In addition, there was the study that showed men carriers of the CYP2C9 mutant genotype (rs1057910) seem to have a lower risk for suffering MI in Austria [35]. There was also the research which suggested CYP2C9 gene interaction with smoking was associated with CAD [36]. In our study, it was not found that rs1057910 of CYP2C9 gene was associated with the risk of CAD. There might be ethnic and geographical environment factors explaining the difference among clinical trials.

## Conclusion

In conclusion, CC genotype of rs2475376 and C-C-A-T haplotype in CYP2C9 may be a risk genetic marker of CAD in women. T allele of rs2475376, the haplotype (C-T-A-C) and the diplotype (CTAC/CTAC) could be protective genetic markers of CAD for women in Han population of China.

## References

[1] Manace LC, Godiwala TN, Babyatsky MW (2009). Genomics of cardiovascular disease. Mt Sinai J Med.

[2] Damani SB, Topol EJ (2011). Emerging genomic applications in coronary artery disease. JACC Cardiovasc.

[3] Herrington DM (2010). Cardiovascular genomics: outcomes and implications. Can J Cardiol.

[4] Lee RL, Goldstein JA, Pieper JA (2002). Cytochrome P450 2C9 polymorphisms: a comprehensive review of the in-vitro and human data. Pharmacogenetics.

[5] Fleming I (2001). Cytochrome p450 and vascular homeostasis. Circ Res.

[6] Fisslthaler B, Popp R, Kiss L, Potente M, Harder DR, Fleming I, Busse R (1999). Cytochrome P450 2C is an EDHF synthase in coronary arteries. Nature.

[7] Delozier TC, Kissling GE, Coulter SJ, Dai D, Foley JF, Bradbury JA, Murphy E, Steenbergen C, Zeldin DC, Goldstein JA (2007). Detection of Human CYP2C8, CYP2C9 and CYP2J2 in Cardiovascular Tissues Drug. Metab Dispos.

[8] Dorado P, Beltrán LJ, Machín E, Peñas-Lledó EM, Terán E, Llerena A, CEIBA.FP Consortium of the Ibero-American Network of Pharmacogenetics and Pharmacogenomics RIBEF (2012). Losartan hydroxylation phenotype in an Ecuadorian population: influence of CYP2C9 genetic polymorphism, habits and gender. Pharmacogenomics.

[9] Nguyen N, Anley P, Yu MY, Zhang G, Thompson AA, Jennings LJ (2012). Genetic and Clinical Determinants Influencing Warfarin Dosing in Children with Heart Disease. Pediatr Cardiol.

[10] Zeldin DC (2001). Epoxygenase pathways of arachidonic acid metabolism. J Biol Chem.

[11] Zordoky BNM, El-Kadi AOS (2010). Effect of cytochrome P450 polymorphism on arachidonic acid metabolism and their impact on cardiovascular diseases. Pharmacol Therapeut.

[12] Spiecker M, Liao JK (2005). Vascular protective effects of cytochrome p450 epoxygenase derived eicosanoids. Arch Biochem Biophys.

[13] Fleming I, Busse R (2006). Endothelium-derived epoxyeicosatrienoic acids and vascular function. Hypertension.

[14] Spector AA, Gary D, Snyder NL (2004). Epoxyeicosatrienoic acids (EETs): metabolism and biochemical function. Progr Lipid Res.

[15] Yousif MH, Benter IF, Roman RJ (2009). Cytochrome P450 metabolites of arachidonic acid play a role in the enhanced cardiac dysfunction in diabetic rats following ischaemic reperfusion injury. Auton Autacoid Pharmacol.

[16] Nithipatikom K, Moore JM, Isbell MA, Falck JR, Gross GJ (2006). Epoxyeicosatrienoic acids in cardioprotection: ischemic versus reperfusion injury. Am J Physiol Heart Circ Physiol.

[17] Kaur-Knudsen D, Bojesen SE, Nordestgaard BG (2009). Common polymorphisms in CYP2C9, sub-clinical atherosclerosis and risk of ischemic vascular disease in 52000 individuals. Pharmacogenomics J.

[18] Haschke-Becher E, Kirchheiner J, Trummer O, Grünbacher G, Kainz A, Boehm BO, März W, Renner W (2010). Impact of CYP2C8 and 2C9 polymorphisms on coronary artery disease and myocardial infarction in the LURIC cohort. Pharmacogenomics.

[19] Shi YY, He L (2005). SHEsis, a powerful software platform for analyses of linkage disequilibrium, haplotype construction, and genetic association at polymorphism loci. Cell Res.

[20] Li Z, Zhang Z, He Z, Tang W, Li T, Zeng Z, He L, Shi Y (2009). A partition-ligation-combination-subdivision EM algorithm for haplotype inference with multiallelic markers. Cell Res.

[21] Abecasis GR, Altshuler D, Auton A, Brooks LD, Durbin RM, Gibbs RA, Hurles ME, McVean GA, 1000 Genomes Project Consortium (2010). A map of human genome variation from population-scale sequencing. Nature.

[22] Daly MJ, Rioux JD, Schaffner SF, Hudson TJ, Lander ES (2001). High-resolution haplotype structure in the human genome. Nat Genet.

[23] Harder DR, Alkayed NJ, Lange AR, Gebremedhin D, Roman RJ (1998). Functional hyperemia in the brain: hypothesis for astrocyte-derived vasodilator metabolites. Stroke.

[24] Harder DR, Gebremedhin D, Narayanan J, Jefcoat C, Falck JR, Campbell WB, Campbell WB, Roman R (1994). Formation and action of a P4504A metabolite of arachidonic acid in cat cerebral microvessels. Am J Physiol.

[25] Node K, Huo Y, Ruan X, Yang B, Spiecker M, Ley K, Zeldin DC, Liao JK (1999). Anti-inflammatory properties of cytochrome P450 epoxygenase-derived eicosanoids. Science.

[26] Jiang H, McGiff JC, Quilley J, Sacerdoti D, Reddy LM, Falck JR, Zhang F, Lerea KM, Wong PY (2004). Identification of 5, 6-trans-epoxyeicosatrienoic acid in the phospholipids of red blood cells. J Biol Chem.

[27] Harris RC, Munger KA, Badr KF, Takahashi K (1990). Mediation of renal vascular effects of epidermal growth factor by arachidonate metabolites. Faseb J.

[28] Dos Santos EA, Dahly-Vernon AJ, Hoagland KM, Roman RJ (2004). Inhibition of the formation of EETs and 20-hete with 1-aminobenzotriazole attenuates pressure natriuresis. Am J Physiol Regul Integr Comp Physiol.

[29] Kondo T, Hirose M, Kageyama K (2009). Roles of oxidative stress and redox regulation in atherosclerosis. J Atheroscler Thromb.

[30] Chaudhary KR, Zordoky BN, Edin ML, Alsaleh N, El-Kadi AO, Zeldin DC, Seubert JM (2012). Differential effects of soluble epoxide hydrolase inhibition and CYP2J2 overexpression on postischemic cardiac function in aged mice. Prostag Other Lipid Mediat Prostag Other Lipid Mediat.

[31] Zhao TT, Wasti B, Xu DY, Shen L, Du JQ, Zhao SP (2012). Soluble epoxide hydrolase and ischemic cardiomyopathy. Int J Cardiology.

[32] Fleming I, Michaelis UR, Bredenkotter D, Fisslthaler B, Dehghani F, Brandes RP, Busse R (2001). Endothelium-derived hyperpolarizing factor synthase (cytochrome P450 2C9) is a functionally significant source of reactive oxygen species in coronary arteries. Circ Res.

[33] Yasar U, Bennet AM, Eliasson E, Lundgren S, Wiman B, De Faire U, Rane A (2003). Allelic variants of cytochromes P450 2C modify the risk for acute myocardial infarction. Pharmacogenetics.

[34] Visser LE, Schaik RH, Jan Danser AH, Hofman A, Witteman JC, Van Duijn CM, Uitterlinden AG, Pols HA, Stricker BH (2007). The risk of myocardial infarction in patients with reduced activity of cytochrome P450 2C9. Pharmacogenet. Genomics.

[35] Funk M, Endler G, Freitag R, Wojta J, Huber K, Mannhalter C, Sunder-Plassmann R (2004). CYP2C9*2 and CYP2C9*3 alleles confer a lower risk for myocardial infarction. Clin Chem.

[36] Ercan B, Ayaz L, Cicek D, Tamer L (2008). Role of CYP2C9 and CYP2C19 polymorphisms in patients with atherosclerosis. Cell Biochem Funct.

